# In-hospital stroke protocol outcomes before and after the implementation of neurological assessments by telemedicine: an observational case–control study

**DOI:** 10.3389/fneur.2024.1303995

**Published:** 2024-03-05

**Authors:** Rodrigo Meirelles Massaud, Tarso Augusto Duenhas Accorsi, Cristina Gonçalves Massant, Gisele Sampaio Silva, Anna Verena de Carvalho Leite, Marcelo Franken, Flavio Tocci Moreira, Karen Francine Köhler, Karine De Amicis Lima, Renata Albaladejo Morbeck, Carlos Henrique Sartorato Pedrotti

**Affiliations:** ^1^Department of Telemedicine, Hospital Israelita Albert Einstein, São Paulo, Brazil; ^2^Clinical Practices Management, Hospital Israelita Albert Einstein, São Paulo, Brazil

**Keywords:** telemedicine, stroke, thrombolytic therapy, mechanical thrombectomy, access to health services

## Abstract

**Purpose:**

Stroke is the second leading cause of global adult mortality and the primary cause of disability. A rapid assessment by a neurologist for general and reperfusion treatments in ischemic strokes is linked to decreased mortality and disability. Telestroke assessment is a strategy that allows for neurological consultations with experienced professionals, even in remote emergency contexts. No randomized studies have compared face-to-face neurological care outcomes with telestroke care. Whether neurologists in an institution achieve better results remotely than in person is also unknown. This study aimed to compare mortality and other outcomes commonly measured in stroke protocols for stroke patients assessed by a neurologist via face-to-face evaluations and telestroke assessment.

**Methods:**

Observational single-center retrospective study from August/2009 to February/2022, enrolling 2,689 patients with ischemic stroke, subarachnoid hemorrhage, and intracerebral hemorrhage. Group 1 (G1) comprised 2,437 patients with in-person neurological assessments, and Telemedicine Group 2 (G2) included 252 patients.

**Results:**

The in-person group had higher admission NIHSS scores (G1, 3 (0; 36) vs. G2, 2 (0; 26), *p* < 0.001). The door-to-groin puncture time was lower in the in-person group than in the telestroke group (G1, 103 (42; 310) vs. G2, 151 (109; 340), *p* < 0.001). The telestroke group showed superior metrics for door-to-imaging time, symptomatic hemorrhagic transformation rate in ischemic stroke patients treated with intravenous thrombolysis, hospital stay duration, higher rates of intravenous thrombolysis and mechanical thrombectomy, and lower mortality. Symptomatic hemorrhagic transformation rate was smaller in the group evaluated via telestroke (G1, 5.1% vs. G2, 1.1%, *p* = 0.016). Intravenous thrombolysis and mechanical thrombectomy rates were significantly higher in telestroke group: (G1, 8.6% vs. G2, 18.2%, *p* < 0.001 and G1, 5.1% vs. G2, 10.4%, *p* = 0.002, respectively). Mortality was lower in the telestroke group than in the in-person group (G1, 11.1% vs. G2, 6.7%, *p* = 0.001). The percentage of patients with an mRS score of 0–2 at discharge was similar in both groups when adjusting for NIHSS score and age.

**Conclusion:**

The same neurological emergency team may assess stroke patients in-person or by telemedicine, with excellent outcome metrics. This study reaffirms telestroke as a safe tool in acute stroke care.

## 1 Introduction

Stroke is the second leading cause of death among adults worldwide and the leading cause of disability (1). Ischemic stroke is caused by acute arterial occlusion and is responsible for most cases of stroke (2). A rapid assessment by a neurologist aimed at managing reperfusion strategies, either through intravenous thrombolysis (IV) or endovascular thrombectomy, is associated with reduced stroke mortality and disability (3). A managed stroke protocol implies that institutional strategies exist to ensure early and adequate assessment of suspected cases, with proper treatment for optimized clinical outcomes (3).

The rapid evaluation of the clinical presentation, request and interpretation of imaging tests, and the provision of guidance for adequate reperfusion treatment are usually performed by a neurologist (4, 5). The availability of a neurologist 24/7 in emergency facilities is complex and has implications for cost-effectiveness (6). Assessment by a neurologist via telemedicine (TM) is a strategy that allows for neurological consultations with experienced professionals, even in remote emergency contexts (7). This approach has been used for approximately two decades, and several emergency departments already provide cameras and are connected to neurological centers for remote neurological consultation in stroke cases (8). Presumably, remote neurological assessment can improve the care of a patient with suspected stroke initially provided by a general practitioner, increasing diagnostic speed and accuracy and initiating a reperfusion strategy (9).

There is much evidence showing that remote assessment by a neurologist is associated with improvement in triage, an increase in the number of reperfusion treatments, a reduction in time for the administration of thrombolytics, an increase in the number of endovascular treatments, optimization of referrals to higher-level hospitals and improvement in prognosis when compared to usual therapy by emergency physicians (10–13).

To establish a telestroke service as the standard of care in hospitals with low stroke admission rates and limited therapeutic resources is always a challenge. The American Academy of Neurology outlines a comprehensive training curriculum for healthcare professionals and neurology residents in fundamental topics: introduction to technology and basic implementation, legal and ethical aspects in teleneurology, developing a caring attitude (empathetic attitude), and teaching specific clinical skills for teleneurology (14). The implementation process would involve training healthcare professionals and neurology residents in these essential topics to ensure a standardized approach to teleneurology. This would enable hospitals with limited stroke resources to provide timely and high-quality stroke care through telemedicine, bridging the gap in stroke management. By emphasizing technology, ethics, communication, and clinical skills development, the curriculum can equip healthcare teams in these hospitals to effectively assess and manage stroke patients remotely, even in settings with constrained resources (15). This approach can help improve patient outcomes and ensure that stroke care meets the highest standards, even in hospitals with small stroke admission volumes and limited therapeutic capabilities.

The absence of randomized studies comparing the outcomes of traditional, face-to-face care (considered the gold standard in stroke management) with those of telestroke care underscores a significant gap in our understanding of stroke treatment efficacy. This gap is particularly relevant as the deployment of Telestroke services varies widely across different national and local settings, suggesting a need for more nuanced research into its efficacy, performance, and benefits in diverse healthcare environments. We hypothesize that there will be no significant difference in mortality and clinical outcomes between acute stroke patients evaluated by the same stroke team in person and those assessed through telestroke. This hypothesis challenges the prevailing assumption that in-person evaluation inherently results in better patient outcomes and addresses the practical question of whether telestroke can stand as a viable standard of care in settings with varying levels of access to specialized stroke care. By examining this, our study not only contributes to the body of evidence supporting telestroke as a potentially equivalent alternative to face-to-face consultation but also informs policy and practice in the deployment of telemedicine services for stroke care, particularly in underserved or resource-limited environments. Therefore, this study aimed to compare mortality, and other performance indicators of an acute stroke protocol (the modified Rankin scale score at discharge, door-to-imaging time, door-to-needle time (IV thrombolysis), door-to-groin puncture time) in patients with ischemic stroke treated with mechanical thrombectomy, symptomatic hemorrhagic transformation rate in patients with ischemic stroke treated with intravenous thrombolysis, and length of hospital stay in stroke patients assessed by a neurologist via face-to-face evaluations and remotely via telestroke assessment.

## 2 Materials and methods

This study was conducted at Hospital Israelita Albert Einstein, a 620-bed private general hospital in São Paulo, Brazil. The hospital adopted a managed stroke care protocol, monitoring quality indicators and establishing actions for continuous improvement.

This was a single-center study with an observational retrospective study design. The stroke team neurologists, involved in patient care 24 h a day, 7 days a week, did not participate in data collection or storage. This study, named TeleStroke, was approved by the Institutional Ethics Committee under registration number CAAE: 58048422.9.0000.0071 and protocol number 5.400.787. All data can be accessed in the deidentified database maintained by the data management group. The protocol followed the hospital’s ethical standards set by the Ethics Committee for Analysis of Research Projects on Human Experimentation.

We included in the analysis patients over 18 years of age with a confirmed diagnosis of acute stroke. All acute onset strokes were included in the sample. Patients confirmed with stroke mimics were excluded from the analysis.

The emergency system encompasses four strategically located emergency care units throughout the city, referred to as “emergency satellite units” (spoke hospitals), in addition to the emergency unit situated at the main hospital (the hub).

São Paulo, being one of the world’s largest metropolitan regions, faces significant urban mobility challenges, largely attributed to traffic congestion. The implementation of multiple emergency satellite units across the city serves the strategic purpose of extending emergency care services to regions geographically distant from the main hospital. This approach is instrumental in facilitating faster and more efficient access to critical healthcare services.

**Figure 1:**
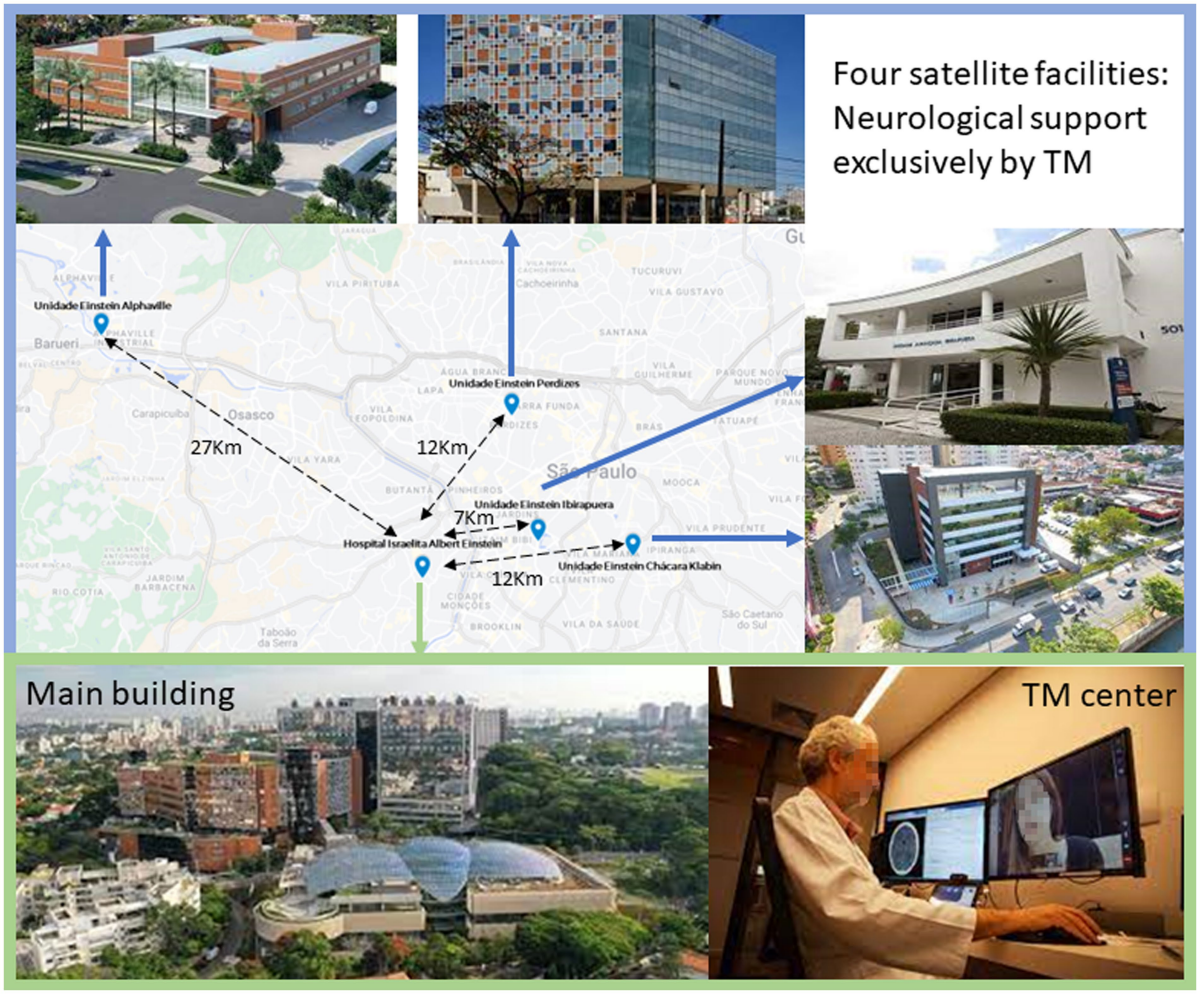
Main building, satellite facilities, and the Telemedicine Center. TM, telemedicine.

In our telemedicine system, we employ a approach to facilitate remote medical consultations between our Hub Hospital and Spoke Hospitals, ensuring seamless communication and efficient patient care. At the core of our telemedicine infrastructure is the utilization of a mobile cart stationed at the patient’s bedside in spoke hospitals, allowing for real-time teleconsultation (Figure 1).

At the Hub Hospital, stroke neurologists are equipped with a speaker, keyboard, mouse and a desktop, coupled with a webcam to provide visual access to the patient’s. The Spoke Hospitals are equipped with a mobile cart specifically designed for telemedicine purposes. This mobile cart encompasses essential hardware and software resources, including a powerful microprocessor, a user-friendly keyboard, a responsive mouse, an ultra-HD or Pan-Tilt-Zoom (PTZ) camera for high-quality visual assessment, and a speaker for clear audio communication. Furthermore, the mobile cart is fortified with an uninterruptible power supply (UPS) to ensure continuous operation even during power fluctuations or outages.

The healthcare providers at the Hub hospital and the Spoke Hospitals have access to critical resources, such as the web-based Picture Archiving and Communication System (PACS) for comprehensive image analysis and the institutional electronic health records stored within the Cerner Millennium platform. Additionally, our system is powered by the sophisticated Software for Electronic Health Record (EHR) – the Einstein Telemedicine System (ETS), which enables comprehensive patient data management and streamlined telemedicine workflows. To facilitate teleconferencing, we employ the Telemedicine Einstein video conferencing software, ensuring real-time, secure, and efficient communication.

Within the main hospital, a team of vascular neurologists is on continuous standby within the telemedicine department.

Stroke team neurologists and emergency physicians receive annually training, based on the best scientific evidence, in the diagnosis and treatment of neurological emergencies. All physicians involved in stroke care are certified in the application of the NIH stroke scale and receive specific training to provide telemedicine and telestroke care in accordance with the recommendations of the American Academy of Neurology.

The hospital’s telemedicine system is activated whenever a nurse suspects a potential stroke. All hospital nurses are trained and certified every year to apply the NIH stroke scale and LAPSS screen. Upon suspicion, the nurse at the spoke hospitals, administers the Los Angeles Prehospital Stroke Screen (LAPSS) to evaluate the patient’s condition. If the LAPSS indicates compatibility with a stroke, the telemedicine system is promptly triggered. This activation process ensures the expeditious provision of specialized stroke care to the patient, thereby optimizing the assessment and treatment of potential stroke cases through the hospital’s telemedicine infrastructure. Patients admitted to satellite units with suspected strokes (LAPSS positive) undergo telestroke evaluations. If intravenous thrombolysis is indicated, thrombolytic treatment is administered within the admission unit, aligning with the “drip and ship” model adopted by emergency satellite facilities. Advanced endovascular treatment resources and dedicated neurological intensive care units are exclusively available at the main hospital. Consequently, all patients admitted to satellite units with suspected strokes are subsequently transferred to the main hospital, and if necessary, referred for mechanical thrombectomy and or intensive neurological care. Patients admitted to the main hospital’s emergency unit or in-hospital strokes receive in-person evaluations by the stroke team.

### 2.1 Outcomes

Outcomes analysis includes in-hospital mortality, modified Rankin scale score at discharge, door-to-imaging time, door-to-needle time (in IV thrombolysis), door-to-groin puncture time in patients treated with mechanical thrombectomy, symptomatic hemorrhagic transformation rate in patients with ischemic stroke treated with intravenous thrombolysis, and length of hospital stay.

### 2.2 Statistical analysis

We used a convenience sample of all consecutive registered stroke patients following our institutional stroke protocol. Continuous variables are expressed as the means and standard deviations or medians and quartiles, and categorical variables are presented as counts and percentages. The Kolmogorov–Smirnov test was used to evaluate the distribution of the sample. We used Student’s *t*-test to compare continuous variables that followed a normal distribution, the Mann–Whitney test for non-normally-distributed continuous variables, and the chi-square test for categorical variables. Additionally, we performed a multivariate logistic regression analysis. Patients’ hospital discharge times were estimated according to the qualitative characteristics of interest using the Kaplan–Meier function, and discharge times were compared using log-rank tests. The unadjusted hazard ratios (HRs) were estimated using bivariate Cox regression, and the joint model with all the characteristics of interest was adjusted using multiple Cox regression. Values with *p* < 0.05 were considered statistically significant, and a 95% confidence interval was established. IBM-SPSS for Windows version 22.0 software was used for statistical calculations.

## 3 Results

### 3.1 Patients

From August 27, 2009, to February 10, 2022, 2,689 patients were included for analysis. A total of 2,437 patients who underwent in-person neurological assessments were included in Group 1 (G1), while 252 patients who were evaluated by telestroke assessment were included in Group 2 (G2). The in-person population was older than the telestroke assessment population (G1, 69.5 ± 17.3 years vs. G2, 63.5 ± 17.7 years, *p* < 0.001) (Table 1).

**Table 1:** Demographic data, clinical characteristics and outcomes.

Variable	Group		Total	*p*
	In-Person	TM		
**Age (years)**	**(*N* = 2,437)**	**(*N* = 252)**	**(*N* = 2,689)**	<0.001**
Mean ± SD	69.5 ± 17.3	63.5 ± 17.7	68.9 ± 17.4	
**Admission NIHSS score**	**(*N* = 1,999)**	**(*N* = 208)**	**(*N* = 2,207)**	**<0.001** ^£^
Median (min.; max.)	3 (0; 36)	2 (0; 26)	3 (0; 36)	
**Door-to-imaging time all strokes (min.)**	**(*N* = 1,379)**	**(*N* = 156)**	**(*N* = 1,535)**	**<0.001** ^£^
Median (min.; max.)	44 (0; 1,423)	38 (16; 445)	43 (0; 1,423)	
**Door-to-needle time (IV trombolysis) in ischemic stroke (min.)**	**(*N* = 164)**	**(*N* = 35)**	**(*N* = 199)**	0.181^£^
Median (min.; max.)	53 (13; 198)	57 (30; 178)	54 (13; 198)	
**Door-to-groin puncture time in ischemic stroke (min.)**	**(*N* = 98)**	**(*N* = 20)**	**(*N* = 118)**	**<0.001** ^£^
Median (min.; max.)	103.5 (42; 310)	151.5 (109; 340)	113 (42; 340)	
**Modified Rankin scale score at discharge, all patients (mrs score)**				**0.028** ^†^
0	845 (35.5)	103 (43.1)	948 (36.2)	
1	421 (17.7)	35 (14.6)	456 (17.4)	
2	125 (5.3)	10 (4.2)	135 (5.2)	
3	137 (5.8)	12 (5)	149 (5.7)	
4	376 (15.8)	45 (18.8)	421 (16.1)	
5	213 (8.9)	18 (7.5)	231 (8.8)	
6	263 (11.1)	16 (6.7)	279 (10.7)	
**Final diagnosis, n (%)**				**0.001** ^†^
SAH	184 (7.6)	35 (13.9)	219 (8.1)	
IS	1,913 (78.5)	192 (76.2)	2,105 (78.3)	
ICH		25 (9.9)	365 (13.6)	
**Hemorrhagic transformation, n (%)**				**0.016** ^†^
No	1,742 (94.9)	179 (98.9)	1,921 (95.3)	
Yes	93 (5.1)	2 (1.1)	95 (4.7)	
**Outcome, n (%)**				**0.001** ^†^
Death	263 (11.1)	16 (6.7)	279 (10.7)	
**Total length of stay**	**(*N* = 2,437)**	**(*N* = 252)**	**(*N* = 2,689)**	**0.001** ^£^
Median (min.; max.)	8 (0; 593)	6 (0; 166)	7 (0; 593)	

^†^Chi-square test; ** Student’s *t*-test; ^£^ Mann–Whitney Test; G1, Group 1; G2, Group 2; IV, intravenous; SAH, subarachnoid hemorrhage; IS, ischemic stroke; ICH, hemorrhagic stroke. *n* = number of patients.

### 3.2 Outcomes

The in-person group had higher admission NIHSS scores (G1, 3 (0; 36) vs. G2, 2 (0; 26), *p* < 0.001). The door-to-groin puncture time was lower in the in-person group than in the telestroke group (G1, 103 (42; 310) vs. G2, 151 (109; 340), *p* < 0.001). The telestroke group had better metrics regarding door-to-imaging time, symptomatic hemorrhagic transformation rate in patients with ischemic stroke (IS) treated with intravenous thrombolysis, length of hospital stay, lower mortality (Table 1).

The telestroke group had higher rates of intravenous thrombolysis and mechanical thrombectomy (Table 2). Symptomatic hemorrhagic transformation rate in patients with ischemic stroke, treated with intravenous thrombolysis, was smaller in the group evaluated via telestroke (G1, 5.1% vs. G2, 1.1%, *p* = 0.016). Intravenous thrombolysis rates were significantly higher in the group evaluated by telestroke (G1, 8.6% vs. G2, 18.2%, *p* < 0.001). Mechanical thrombectomy rates were significantly higher in the group evaluated by telestroke (G1, 5.1% vs. G2, 10.4%, *p* = 0.002). Mortality was lower in the telestroke group than in the in-person group (G1, 11.1% vs. G2, 6.7%, *p* = 0.001).

**Table 2:** Reperfusion treatment according to patient assessment strategy.

Variable	Group		Total	*p*
	In-Person	TM		
IV thrombolysis, n (%)				**<0.001**
No	1,749 (91.4)	157 (81.8)	1,906 (90.5)	
Yes	164 (8.6)	35 (18.2)	199 (9.5)	
Mechanical thrombectomy, n (%)				**0.002**
No	1,815 (94.9)	172 (89.6)	1,987 (94.4)	
Yes	98 (5.1)	20 (10.4)	118 (5.6)	

Chi-square test, *n* = number of patient.

The number of IS was similar, but the subarachnoid hemorrhage (SAH) number was higher in the group evaluated by telestroke, and the hemorrhagic stroke (ICH) number was higher in the person group. The joint analysis of final diagnoses shows significant differences.

The percentage of patients with an mRS score of 0–2 at discharge was similar in both groups when adjusting for NIHSS score and age.

The door-to-needle time in ischemic stroke patients was not significantly different between the two groups, and the median is by the goals proposed by Get with the Guidelines from the American Stroke Association.

## 4 Discussion

This study compared outcomes in stroke patients with an in-person assessment versus telestroke assessment performed by the same team of neurologists at the same institution. Despite the nonrandomized and unicentric design, we analyzed a representative number of patients treated in a hospital with a stroke protocol. Our results suggested similar outcomes between the telestroke and in-person groups.

Health care costs associated with acute stroke is predicted to increase, reflecting aging and chronically ill American and Brazilian populations (16–18). Telehealth has become a significant and critical component of health care delivery. In 2017, the American Hospital Association reported that 65% of hospitals in the United States of America connected with patients and consulting practitioners using video and other technology, which has grown exponentially since the COVID-19 pandemic (18).

Neurological telestroke emergency evaluations have several benefits, including better access to care for patients, greater efficiency in the delivery of health care (19), and improved cost-effectiveness (11, 20, 21). The STROKEDOC (Stroke Team Remote Evaluation Using a Digital Observation Camera) pooled analysis supported the hypothesis that compared with telephone-only consultations, TM consultations, which included teleradiology consultations, resulted in significantly more accurate decision-making regarding intravenous alteplase eligibility for patients exhibiting symptoms and signs of an acute stroke in the emergency department (22). Therefore, the use of TM resources and systems should be supported by health care institutions, governments, payers, and vendors as a method to ensure adequate 24/7 coverage and care for acute stroke patients in various settings (23–26).

Ho et al. described developing a local telestroke system that allows stroke neurologists to access and treat patients via telemedicine. The trigger for this methodology was the COVID-19 pandemic. After calling the stroke code, the responsible neurologist decided whether the patient would be seen in person or virtually. For telestroke evaluation, the neurologist assessed the patient with the respective devices in the company of an emergency department nurse, and the emergency doctor was available if necessary (27). A total of 55 patients were assessed by telemedicine, and the outcomes were similar to the in person evaluation. This study was the first to evaluate stroke outcomes with in-person and telemedicine care, carried out by the same team of neurologists. Our study presents some differences: the program was installed before the pandemic, emergency doctors and nurses monitored patients, and we analyzed a more significant number of patients and more outcomes. Almalloouhi et al. compared outcomes in patients who receive rPA at a spoke site and remain there for post-tPA care by telemedicine with patients treated at a comprehensive stroke center with in-person care. The scope is different but demonstrates telemedicine’s importance and non-inferiority of neurological assessment (28).

In the recent study of Alexandra et al., the Telestroke program was implemented in 2018. Sixty-five patients were analyzed in person (NIHSS 11) and 35 by telemedicine (NIHSS 12). The average onset of symptoms was 136 min in face -to -face thrombolysis and 81 min in the Telestroke model, *p* = 0.0083. In discharge, both groups had a median NIHSS score of 4. The authors concluded that thrombolysis therapy was safe and effective in both strategies (29). Our findings of better protocol metrics in the telestroke group were surprising. This could be partly attributed to the younger age and lower NIHSS scores in the telestroke group, facilitating faster and easier neurological evaluations. We draw attention to higher rates of patients with ischemic stroke who received intravenous thrombolytic and mechanical thrombectomy in the group evaluated via telestroke. Despite endovascular treatment being only available in the hospital, patients admitted to emergency satellite units had higher mechanical thrombectomy rates, even though they were permanently transferred to the hospital to receive this treatment. The ambulance transfer to the hospital probably explains the longer door-to-groin puncture times in the telestroke group. Despite longer door-to-groin puncture times, there is no difference in rates of patients with mRS scores of 0–2 between the two groups. The implementation of Telestroke systems might mitigate main hospital overload, optimizing patient screening and treatment. Specifically, in our sample, the Telestroke group exhibited superior outcomes, including reduced door-to-imaging times, higher rates of intravenous thrombolysis and mechanical thrombectomy, lower rates of symptomatic hemorrhage, and reduced mortality. These findings raise the hypothesis that telemedicine could significantly enhance the efficiency and effectiveness of stroke care protocols, serving as a valuable extension of neurological care in emergencies and potentially leading to substantial improvements in patient outcomes.

The main limitation of our study was its retrospective design, which resulted in the unavailability of certain variables, such as the lesion volume, vessel occlusion site, and comorbidities. While our ethical committees exempted us from obtaining informed consent, we were restricted from accessing sensitive variables. However, the analyzed outcome measures could be compared without any missing data. Importantly, our institutional stroke protocol ensures the confirmability and accuracy of the collected data.

## 5 Conclusion

In conclusion, in-person and telestroke assessments by the same neurological emergency team demonstrated excellent outcome metrics in stroke patients at the same center. This study confirms that telestroke assessments are a safe and effective tool in acute stroke care. Prospective studies aiming for cost-effectiveness should be encouraged for optimizing health systems, especially in developing countries.

## Data availability statement

The raw data supporting the conclusions of this article will be made available by the authors, without undue reservation.

## Ethics statement

The studies involving humans were approved by Hospital Israelita Albert Einstein ethics committee/institutional review board. The studies were conducted in accordance with the local legislation and institutional requirements. The ethics committee/institutional review board waived the requirement of written informed consent for participation from the participants or the participants’ legal guardians/next of kin because it was a retrospective study reviewing patient data.

## Author contributions

RMa: Conceptualization, Data curation, Formal analysis, Writing – original draft. TA: Conceptualization, Formal analysis, Writing – original draft, Writing – review & editing. CM: Conceptualization, Data curation, Writing – review & editing. GS: Conceptualization, Data curation, Formal analysis, Writing – review & editing. AdC: Conceptualization, Data curation, Writing – review & editing. MF: Conceptualization, Writing – review & editing. FM: Writing – review & editing. KK: Project administration, Writing – review & editing. KD: Writing – review & editing, Project administration. RMo: Supervision, Writing – review & editing. CP: Supervision, Writing – review & editing.
